# Supplementary material for: Splice-Junction-Based Mapping of Alternative Isoforms in the Human Proteome
Source: Cell Rep. Author manuscript; Available in PMC 2020 Jan 15. (PMC6961840; doi:10.1016/j.celrep.2019.11.026)
Supplement: 2 [file NIHMS1546469-supplement-2.zip › DF1/PXD010154/Testis/Testis_33_EPB41L3_QEDAPMIEPLVPEEVSSQK.pdf]

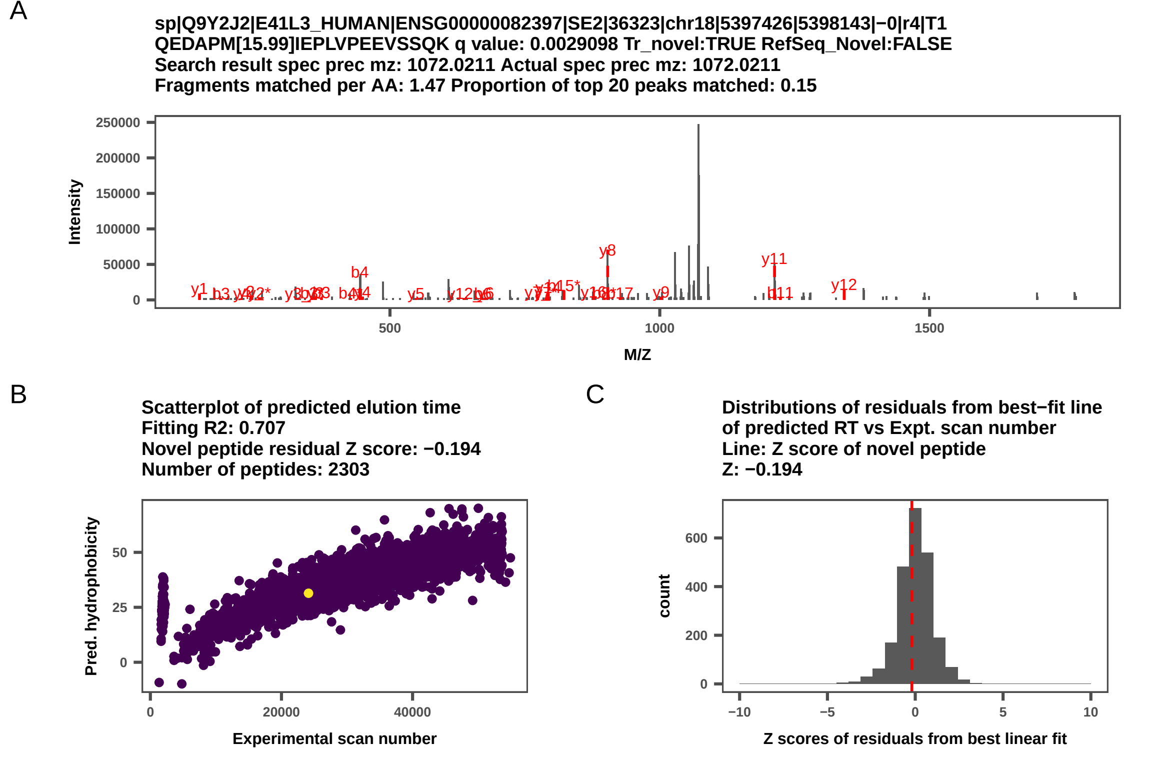
A
sp|Q9Y2J2|E41L3_HUMAN|ENSG00000082397|SE2|36323|chr18|5397426|5398143|−0|r4|T1
QEDAPM[15.99]IEPLVPEEVSSQK q value: 0.0029098 Tr_novel:TRUE RefSeq_Novel:FALSE
Search result spec prec mz: 1072.0211 Actual spec prec mz: 1072.0211
Fragments matched per AA: 1.47 Proportion of top 20 peaks matched: 0.15
Intensity
250000
200000
150000
100000
50000
0
y1
b3
b4
y5
y8
y9
y11
b11
y12
500
1000
1500
M/Z
B
Scatterplot of predicted elution time
Fitting R2: 0.707
Novel peptide residual Z score: −0.194
Number of peptides: 2303
Pred. hydrophobicity
50
25
0
0
20000
40000
Experimental scan number
C
Distributions of residuals from best−fit line
of predicted RT vs Expt. scan number
Line: Z score of novel peptide
Z: −0.194
count
600
400
200
0
−10
−5
0
5
10
Z scores of residuals from best linear fit
